# Predictors of seasonal influenza vaccination among older adults in Thailand

**DOI:** 10.1371/journal.pone.0188422

**Published:** 2017-11-29

**Authors:** Prabda Praphasiri, Darunee Ditsungnoen, Supakit Sirilak, Jarawee Rattanayot, Peera Areerat, Fatimah S. Dawood, Kim A. Lindblade

**Affiliations:** 1 Influenza program, Thailand MOPH-US CDC Collaboration, Nonthaburi, Thailand; 2 Technical Health Office, Thailand Ministry of Public Health, Nonthaburi, Thailand; 3 National Health Security Office, Bangkok, Thailand; 4 Nakhon Phanom Provincial Health Office, Nakhon Phanom, Thailand; 5 Influenza Division, US Centers for Disease Control and Prevention, Atlanta, GA, United States of America; University of Washington, UNITED STATES

## Abstract

**Background:**

In advance of a large influenza vaccine effectiveness (VE) cohort study among older adults in Thailand, we conducted a population-based, cross-sectional survey to measure vaccine coverage and identify factors associated with influenza vaccination among older Thai adults that could bias measures of vaccine effectiveness.

**Method:**

We selected adults ≥65 years using a two-stage, stratified, cluster sampling design. Functional status was assessed using the 10-point Vulnerable Elders Survey (VES); scores ≥3 indicated vulnerability. Questions about attitudes towards vaccination were based on the Health Belief Model. The distance between participants’ households and the nearest vaccination clinic was calculated. Vaccination status was determined using national influenza vaccination registry. Prevalence ratios (PR) and 95% confidence intervals (CIs) were calculated using log-binomial multivariable models accounting for the sampling design.

**Result:**

We enrolled 581 participants, of whom 60% were female, median age was 72 years, 41% had at least one chronic underlying illness, 24% met the criteria for vulnerable, and 23% did not leave the house on a daily basis. Influenza vaccination rate was 34%. In multivariable models, no variable related to functional status was associated with vaccination. The strongest predictors of vaccination were distance to the nearest vaccination center (PR 3.0, 95% CI 1.7–5.1 for participants in the closest quartile compared to the furthest), and high levels of a perception of benefits of influenza vaccination (PR 2.8, 95% CI 1.4–5.6) and cues to action (PR 2.7, 95% CI 1.5–5.1).

**Conclusion:**

Distance to vaccination clinics should be considered in analyses of influenza VE studies in Thailand. Strategies that emphasize benefits of vaccination and encourage physicians to recommend annual influenza vaccination could improve influenza vaccine uptake among older Thai adults. Outreach to more distant and less mobile older adults may also be required to improve influenza vaccination coverage.

## Introduction

Older adults are at increased risk of influenza-associated hospitalizations and deaths compared to younger adults [1–5]. Many countries now routinely vaccinate persons ≥65 years against influenza, and World Health Organization includes older adults as one of the high-risk groups recommended for annual influenza vaccination [5]. To measure vaccine effectiveness (VE) among risk groups for whom the vaccine is already recommended, observational designs such as cohort or test-negative case-control studies, rather than experimental designs, must be used for ethical reasons[6]. However, selection bias, unmeasured confounding and confounding by indication are particular concerns for observational studies of VE in persons ≥65 years due to the potential relationship between vaccination and factors that affect their likelihood of being vaccinated and seeking health care, such as functional status [7–9]. Persons with reduced mobility, or those unable to care for themselves alone, may have a higher risk of hospitalization or death, and be less likely to be vaccinated than persons with fewer functional limitations. Conversely, if persons with chronic diseases are also considered a priority for influenza vaccination, there may be higher rates of vaccination among persons with more disability. In either case, if functional status or other measures of vulnerability are related both to vaccination and adverse health outcomes, VE estimates may be biased if models do not adequately adjust for propensity to be vaccinated [10].

In advance of a large influenza VE cohort study among Thai adults ≥65 years, we conducted a population-based cross-sectional survey to identify demographic, health, geographic, functional, and socio-economic characteristics associated with influenza vaccination. These data will be used to improve the design and analysis of vaccine effectiveness studies and may also be used to improve vaccination coverage among older Thai adults.

## Materials and methods

### Influenza vaccination in Thailand

Free influenza vaccination is provided annually from May-September to high-risk populations in Thailand, including persons ≥65 years, persons with a self-reported chronic illness, pregnant women, persons who have obesity and children 6 months to 2 years old. The total number of people eligible for free influenza vaccination is estimated at >20 million [11], which is approximately 30% of Thailand’s population. Thailand’s National Health Security Organization (NHSO) and Ministry of Public Health (MOPH) purchased 3.5 million doses of influenza vaccine in 2014 for free distribution to high-risk groups on a first-come, first-served basis (personal communication). Estimated coverage in healthy persons ≥65 years was 13% in 2014 (Bureau of General Communicable Diseases, Thailand MOPH).

In Nakhon Phanom province, located in the northeast of Thailand, four of 12 districts were purposively chosen by provincial health authorities for an enhanced vaccine campaign in 2014. Each district was encouraged to develop their own strategies (including increased education and communication programs, mobile outreach clinics and social mobilization using village health volunteers) to increase coverage to 50% in older adults from a baseline of approximately 30% in 2013. An additional 8,823 doses of influenza vaccine, above the 3,177 doses routinely allocated to Nakhon Phanom Province (a total of 12,000 doses), were obtained from NHSO and MOPH to ensure adequate supplies during the campaign. Persons who received influenza vaccine during the campaign (May–July 2014) were recorded in computer databases by their unique 13-digit population identification code.

### Study population

Nakhon Phanom Province had a population of 708,350 in 2012 [12]. We considered all non-institutionalized residents from the four enhanced campaign districts ≥ 65 years as of May 1, 2014, (estimated population 24,088) as the study population. Non-Thai citizens and persons who could not speak Thai and or were unable to provide informed consent were excluded.

### Sample size

The sample size was calculated using OpenEpi (www.OpenEpi.com) as an unmatched case-control study. We assumed 50% vaccination coverage, based on the campaign target, and thus a 1:1 case to control ratio in the sample. Because the prevalence of factors potentially associated with vaccination was unknown in this population, we chose a minimum prevalence of 10% to maximize the sample size. We based sample size calculation on a prevalence ratio of 2.0 to detect larger effects. We assumed a Type I error rate of 5%, 80% power and used a continuity correction [13]. We used a standard design effect of 2.0 to take into account the cluster sampling design. We chose 30 clusters as the optimal balance between cost and precision and estimated a 20% loss for absences, deaths and refusals. The final sample size was rounded up to a number equally divisible by 30, resulting in a sample size of 690 (23 persons per cluster). This sample size would also be sufficient for estimation of vaccination rate +/- 6 percentage-points.

### Sampling procedure

We used a stratified, two-stage cluster sampling scheme with census enumeration areas (EAs) serving as primary sampling units and individuals ≥65 years as secondary sampling units. We stratified 576 EAs in the four campaign districts of Nakhon Phanom Province by district and then categorized municipal areas *(Thesaban)* as urban and non-municipal areas as rural within each district based on Thailand’s administrative structure. To select 30 clusters, we used proportional allocation to select EAs from each stratum, with a minimum of 1 EA in each stratum. A census of all persons ≥65 years was conducted in each selected EA by district health personnel and village health volunteers in June 2014, and persons ≥65 were randomly selected. Interviews were conducted in participants’ homes by trained survey staff.

### Factors associated with vaccination

Basic demographic data were requested from all participants. A list of household assets was analyzed using principal components analysis to generate a wealth index that was categorized into quintiles [14, 15]. We asked participants for their history of chronic diseases, physician visits for any reason in last three months, hospitalizations for any reason in last 12 months, influenza vaccination in previous years, and any side effects to vaccination such as soreness, redness or swelling at injection site, low-grade fever, aches, and allergic reactions.

To measure functional status, we used an index from the Vulnerable Elders Survey (VES), a 13-item questionnaire which uses information on age, self-reported health status, and frailty as determined by participant’s difficulty in performing routine daily tasks; individuals are scored from 0–10, with higher scores indicating greater degrees of vulnerability [16]. Persons with VES scores ≥3 are considered vulnerable, i.e. at greater risk of mortality [17]. We chose VES-13 as it was a simple, function-based and clinically-relevant tool for screening community-dwelling populations, took an average of less than five minutes for a non-clinical personnel to complete, and demonstrated high sensitivity and specificity in detecting functional deficits and risk of mortality in older subjects. We also asked participants to self-report falls in last 6 months, the frequency of leaving home, and degree of memory loss.

Attitudes towards influenza vaccination were assessed through questions based on the Health Belief Model (HBM), which posits that people are likely to adopt preventive behaviors such as vaccination when they perceive they are susceptible to disease, the disease is severe, the preventive behavior is beneficial, barriers are minimal, and in presence of a stimulus, or cue to action, which encourages the behavior [18]. Two questions in the questionnaire focused on perceived susceptibility to influenza (i.e., likelihood of contracting influenza); three on perceived severity of influenza (i.e., likelihood of severe illness); one on perceived benefits of influenza vaccine (i.e., effectiveness at preventing illness); two on perceived barriers to vaccination (i.e., side effects or perceptions that the vaccine is unsafe); and four on cues to action (i.e., recommendations to get vaccinated or exposure to health messages) (S1 Table). Face validity of the questionnaire was pre-tested among persons ≥65 years outside the survey area to make minor adjustments as needed.

### Data management and analysis

The geographic coordinates of participants’ homes were recorded using a global positioning system; distance in kilometers was calculated to the closest facility where influenza vaccination was offered and categorized into quartiles by district, since distances to vaccination clinic varied considerably by the district. Vaccination in 2014 was verified by matching participants’ 13-digit population identification code to the vaccination database. History of previous vaccination was by self-report.

All statistical analyses were performed with SAS version 9.4 (SAS Institute, NC, USA). Frequencies were weighted using the inverse of combined probabilities of selection at the first and second stages and the probability of non-response for each EA. Score method was used to calculate 95% confidence intervals (CIs) for proportions accounting for survey design [19, 20]. Responses to questions pertaining to each HBM construct were summed into scores. Since the number of questions and range of scores were different for each construct, scores were normalized to a mean of 0 and standard deviation of 1, and mean standardized scores with 95% CIs were compared between vaccinated and unvaccinated individuals; statistical significance was determined by non-overlapping CIs. Associations between key demographic and functional status characteristics of participants were measured using a Rao-Scott Chi square, considering the sampling design, with a p-value <0.05 indicating a statistical association.

A generalized estimating equations (GEE) model with a binomial distribution and log link was used to estimate prevalence ratios (PR) and 95% CIs for factors associated with vaccination, taking sampling weights and stratification by district into account. We adjusted for clustering by EA using an exchangeable correlation structure. Two models were fit with variables from univariable analysis with Type III p-values <0.05: model I included only variables related to participants’ demographic characteristics and functional status, while model II included the variables from model I plus HBM constructs with p<0.05 in univariable analyses, which were dichotomized as low and high based on the median value. Prior vaccination was not included in multivariable models because of a strong correlation with current vaccination and the likelihood that predictors would be same for both current and prior vaccination.

### Ethical considerations

Consent forms were translated into Thai, reviewed by a bilingual staff member, and printed in large font. Written consent was obtained by the interviewer from all participants. The survey was approved by the Ethical Review Committee for Research in Human Subjects, Department of Disease Prevention & Control (DDC), Thailand Ministry of Public Health (FWA00013622) and the U.S. Centers for Disease Control and Prevention (CDC), Atlanta, GA (FWA00001413).

## Results

### Demographic characteristics and functional status

Of the 690 persons ≥65 years of age selected, 595 (86%) were enrolled; 14% were lost mostly due to absences, deaths and errors in census data. The final analytic sample comprised 581 (98%) participants who were able to provide their 13-digit population identification code. Participants had a median age of 72 years (interquartile range [IQR], 68–77 years) and 60% were female (Table 1). Few participants (10%) had completed primary school, the majority (54%) earned <5000 baht (156 USD) per month, and almost 100% had health insurance. Median distance from participants’ homes to the closest influenza vaccination clinic was 2.9 km (IQR 1.1–8.2).

**Table 1:** Descriptive characteristics of survey participants by 2014 influenza vaccination status, Thailand*.

Characteristic	All (n = 581)	Vaccinated (n = 216)	Unvaccinated (n = 365)
	n (%)	n (%)	n (%)
Age (years)			
65–74	383 (66)	155 (72)	228 (62)
75–84	164 (28)	56 (26)	108 (30)
>85	33 (6)	5 (2)	28 (8)
Sex, female	346 (60)	127 (59)	219 (60)
Marital status			
Married	272 (47)	122 (56)	150 (41)
Widowed/divorced	286 (49)	87 (40)	199 (55)
Never married	23 (4)	7 (3)	16 (4)
Education			
Never attended school	55 (9)	9 (4)	46 (13)
Attended primary	466 (80)	183 (85)	283 (78)
Completed primary	60 (10)	24 (11)	36 (10)
Average household income per month (USD)			
<156	312 (54)	107 (50)	205 (56)
156–311	103 (18)	43 (20)	60 (16)
>312	110 (19)	44 (20)	66 (18)
Wealth index			
1 (poorest)	114 (20)	30 (14)	84 (23)
2	117 (20)	47 (22)	70 (19)
3	116 (20)	41 (19)	75 (21)
4	116 (20)	45 (21)	71 (19)
5 (least poor)	116 (20)	52 (24)	64 (18)
Has health insurance	579 (100)	216 (100)	363 (99)
District of residence			
Mueang	197 (34)	46 (21)	151 (41)
Plapak	100 (17)	26 (12)	74 (20)
Thatphanom	166 (29)	84 (39)	82 (22)
Srisongkran	118 (20)	60 (28)	58 (16)
Distance from vaccination site			
1 (Nearest)	145 (25)	88 (41)	57 (16)
2	146 (25)	45 (21)	101 (28)
3	144 (25)	67 (31)	77 (21)
4 (Farthest)	146 (25)	16 (7)	130 (36)
Visited a physician in last 3 months	400 (69)	162 (75)	238 (65)
Admitted to hospital in last 12 months	132 (23)	50 (23)	82 (22)
Any chronic disease	236 (41)	99 (46)	137 (38)
Does not leave house on a daily basis	135 (23)	32 (15)	103 (28)
Fell in last 6 months	156 (27)	52 (24)	104 (28)
Moderate to severe memory loss	110 (19)	29 (13)	81 (22)
Vulnerable (VES score ≥3)**	138 (24)	36 (17)	102 (28)
Influenza vaccination			
Previous year	205 (35)	134 (62)	71 (19)
Ever vaccinated	343 (59)	216 (100)	127 (35)
Ever had side effects	57 (10)	32 (15)	25 (7)

*All proportions are weighted according to the survey design and non-response, and are percentages of non-missing data.

USD = United States dollars; VES = Vulnerable Elders Survey [16]

**The VES score could not be calculated for 49 participants.

Visits for any reason to physicians were common with 69% of participants reporting at least one visit in last three months, and 23% of participants had been admitted to hospital in last 12 months (Table 1). History of a chronic illness was reported by 41% of participants. Almost a quarter (23%) of participants did not leave the house on a daily basis, and 27% had fallen in last six months. Median VES score was 1 (IQR, 0–2), and 24% of participants were considered vulnerable (VES≥3). Number and proportion of responses and score components of the modified Vulnerable Elders Survey are presented in S2 Table.

### Associations among demographic and functional status variables

Distance to nearest vaccination clinic was associated with age, income, wealth, memory loss, and vulnerability, but not with a physician’s visit in last 3 months or hospital admission (S3 Table). Vulnerability was associated with sex, marital status, educational level, having a chronic disease, not going out daily, and memory loss.

### Influenza vaccination

In 2014, the survey year, 216 (34%, 95% CI 25–44) participants received an influenza vaccine. The proportion vaccinated varied by district. In the year before the survey, 205 (35%, 95% CI 27–44) participants self-reported receiving an influenza vaccine and 343 (59%, 95% CI 47–66) reported ever having been vaccinated against influenza (Table 1). Most common reasons cited by participants for not getting vaccinated in 2014 were: vaccine was not available (28%); participant did not feel susceptible to influenza (15%); and participant had not considered getting vaccinated (7%) (S4 Table).

### Attitudes towards influenza and vaccination

**Fig 1:**
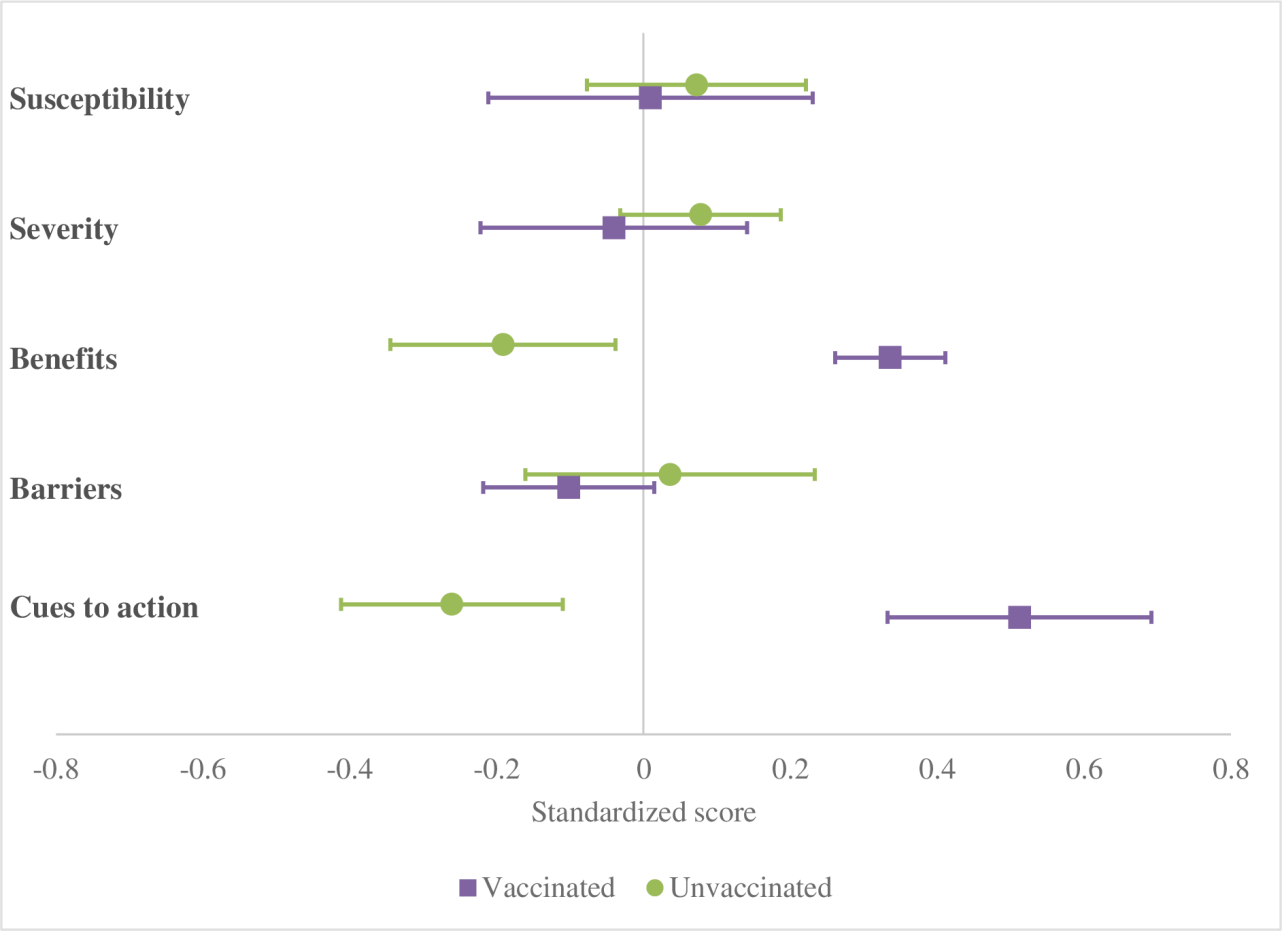
Forest plot of mean standardized scores* and 95% confidence intervals of the Health Belief Model constructs by vaccination status, Thailand (n = 581). *Standardized scores (Z scores) were calculated to account for the different number of items comprising each HBM constructs (S1 Table), such that all constructs of HBM had a mean of 0 and standard deviation of 1.

Vaccinated and unvaccinated participants had overlapping 95% CIs for perceived susceptibility to influenza, severity of influenza, and barriers to vaccination (Fig 1). Vaccinated participants had much higher perceived benefits of vaccination (mean 0.34, 95% CI 0.26–0.41) than those unvaccinated (mean -0.19, 95% CI -0.34 - -0.04). Vaccinated participants also reported higher levels of cues to action (mean 0.51, 95% CI 0.33–0.69) than unvaccinated participants (mean -0.26, 95% CI -0.41 - -0.11).

### Univariable and multivariable analysis

In univariable analyses, factors positively associated with influenza vaccination included younger age, being married, education, resident of Thatphanom District, living nearer to vaccination clinic, visiting a physician in last three months, being vaccinated for influenza in the previous year, high levels of perception of benefits from vaccination and cues to action (Table 2). Factors negatively associated with vaccination for influenza were not leaving house daily and scoring as vulnerable on VES scale. In Model I, which included only variables related to participants’ functional status, being married and living nearer to vaccination clinic remained positively associated, while not leaving the house daily was negatively associated with influenza vaccination. Distance to vaccination clinic was most strongly associated with vaccination; participants in the nearest, second nearest, and third nearest quartile were 3.1 (95% CI, 1.8–5.3), 2.5 (95% CI 1.4–4.3) and 2.5 (95% CI 1.5–4.3) times more likely to get vaccinated, respectively, compared to those in the most distant quartile.

**Table 2:** Factors associated with vaccination among survey participants in 2014 (n = 581).

Characteristic	Univariable p-value **	Multivariable Model I *	Multivariable Model II*
	PR (95% CI)	p-value **	p-value **
		PR (95% CI)	PR (95% CI)
Age (years)	0.0048	0.39	0.7
65–74	2.3 (1.3–4.1)	1.3 (0.7–2.5)	0.9 (0.5–1.6)
75–84	1.7 (0.9–2.9)	1.1 (0.6–2.1)	0.8 (0.4–1.5)
>85	Ref.	Ref.	Ref.
Sex	0.44		
Female	1.1 (0.9–1.4)		
Male	Ref.		
Marital status	<0.0001	0.009	0.33
Married	1.9 (1.1–3.4)	1.7 (0.9–3.3)	1.3 (0.6–2.7)
Widowed/divorced	1.2 (0.6–2.1)	1.2 (0.6–2.4)	1.2 (0.6–2.4)
Never married	Ref.	Ref.	Ref.
Education	0.03	0.06	0.02
None	Ref.	Ref.	Ref.
Primary only	2.0 (1.1–4.0)	1.4 (1.0–1.9)	1.3 (1.0–1.7)
Secondary and above	2.2 (1.2–3.8)	1.3 (1.0–1.7)	1.5 (1.1–2.0)
Average household income per month (USD)	0.44		
<156	Ref.		
156–311	1.0 (0.7–1.4)		
>312	1.2 (0.9–1.5)		
Wealth index	0.05		
1 (poorest)	0.6 (0.4–0.8)		
2	0.8 (0.6–1.1)		
3	0.8 (0.6–1.2)		
4	0.9 (0.6–1.3)		
5 (least poor)	Ref.		
District	0.005		
Mueang	0.9 (0.5–1.5)		
Thatphanom	2.0 (1.2–3.1)		
Srisongkram	1.9 (1.0–3.7)		
Plapak	Ref.		
Distance to vaccination center	<0.0001	0.0002	0.0001
1 (Nearest)	4.0 (2.2–7.4)	3.1 (1.8–5.3)	3.0 (1.7–5.1)
2	2.6 (1.4–4.8)	2.5 (1.4–4.3)	2.4 (1.4–4.2)
3	2.7 (1.5–4.9)	2.5 (1.5–4.3)	2.4 (1.3–4.2)
4 (Farthest)	Ref.	Ref.	Ref.
Visited a physician in last 3 months	0.03	0.07	0.23
Yes	1.4 (1.0–1.8)	1.3 (1.0–1.8)	1.2 (0.9–1.5)
No	Ref.	Ref.	Ref.
Admitted to hospital in last 12 months	0.45		
Yes	0.9 (0.7–1.2)		
No	Ref.		
Any chronic disease	0.33		
Yes	1.2 (0.9–1.6)		
No	Ref.		
Does not leave house daily	0.0002	0.09	0.33
Yes	0.6 (0.5–0.8)	0.8 (0.6–1.0)	0.9 (0.7–1.1)
No	Ref.	Ref.	Ref.
Fell in last 6 months	0.9		
Yes	1.0 (0.8–1.3)		
No	Ref.		
Moderate to severe memory loss	0.19		
Yes	0.8 (0.6–1.1)		
No	Ref.		
Vulnerable	0.0007	0.33	0.16
Yes (VES ≥3)	0.6 (0.5–0.8)	0.9 (0.6–1.2)	0.8 (0.7–1.1)
No (VES <3)	Ref.	Ref.	Ref.
Vaccinated for influenza in previous year	<0.0001		
Yes	3.0 (2.1–4.3)		
No	Ref.		
Perception of susceptibility	0.57		
Low	Ref.		
High	1.1 (0.8–1.4)		
Perception of severity	0.59		
Low	Ref.		
High	1.0 (0.9–1.3)		
Perception of benefits	0.0001		0.005
Low	Ref.		Ref.
High	3.5 (1.8–6.6)		2.8 (1.4–5.6)
Perception of barriers	0.26		
Low	Ref.		
High	0.8 (0.6–1.2)		
Cues to action	<0.0001		0.002
Low	Ref.		Ref.
High	3.0 (1.7–5.2)		2.7 (1.5–5.1)

*Model I includes all variables related to the functional status of the participant with *P*<0.05 in univariable analyses. Model II includes all variables from Model I plus Health Belief Model constructs with *P*<0.05 in univariable analyses. Models I and II contain 531 observations after removing participants with missing VES scores (n = 49) and age (n = 1).

**P values are Type III Wald Statistics from GEE model.

PR = Prevalence Ratio, CI = Confidence Interval.

When HBM constructs were added in Model II, education was positively associated with influenza vaccination, but being married and not leaving house daily were no longer statistically associated. Living nearer to a vaccination clinic remained a strong predictor of vaccination (Table 2). Participants with high levels of perception of benefits from vaccination and cues to action were 2.7 (95% CI 1.4–5.2) and 2.6 (95% CI 1.4–4.9) times more likely to be vaccinated, respectively.

## Discussion

We surveyed community-dwelling Thai adults ≥65 years in Nakhon Phanom Province in 2014 and found that 34% had been vaccinated for influenza after an enhanced vaccination campaign. Influenza vaccine coverage measured in this survey was higher than the estimated 2014 national coverage rate in persons >65 years (13%, Bureau of General Communicable Diseases, Thai MOPH).

Observational studies in older adults could overestimate influenza vaccine effectiveness if healthier adults with fewer functional limitations are more likely to get vaccinated [8, 10]. However, analyses of data from the US in 2000 did not find a significant difference in influenza vaccination rates in older adults by level of disability [21] or mobility [22]. Additionally, studies that incorporated measures of frailty or functional status into multivariable models have not always found them to be confounders of vaccine effectiveness [23, 24]. Conversely, confounding by indication, in which persons with greater disability are more likely to get vaccinated, perhaps as a result of vaccination campaigns targeting those with chronic diseases, could result in an underestimation of vaccine effectiveness [9]. In Thailand, where persons with chronic diseases are included among the target groups for influenza vaccination along with older adults, we believed that either selection bias or confounding by indication was possible. However, in our population-based, cross-sectional survey conducted after an enhanced vaccination campaign, we did not find any association between influenza vaccination and underlying, chronic disease, or any of several measures of functional status in older adults in multivariable models. Studies of vaccine effectiveness in this population are unlikely to be affected by either a healthy or unhealthy vaccinee bias.

Factors most strongly associated with vaccination in multivariable models were distance from vaccination facility, higher educational level, and high levels of perceived benefits of vaccination and cues to action. Distance to health facilities is a known barrier to healthcare [25, 26] but is rarely measured in influenza vaccine effectiveness studies. Thailand is an upper middle income country with free, universal health insurance coverage. Distance from the nearest vaccination clinic was strongly associated with influenza vaccination but was not associated with either a physician’s visit in last three months or hospital admission in last 12 months, suggesting either that distance has a stronger negative impact on preventive than curative behaviors, or that curative medical care may be located closer to participants than vaccination clinics which tend to be centralized at district hospitals rather than at district health centers. These findings suggest strongly that increased outreach efforts, such as mobile units and community networking, are needed to improve the uptake of the vaccine, and perhaps other prevention interventions, among older adults in Thailand.

We did not find perceptions of susceptibility to influenza, severity of illness, or barriers to vaccination to be associated with vaccination. Although older adults are at higher risk for severe complications from influenza, the potential severity of an influenza virus infection did not appear to affect their decision to get vaccinated. However, perceptions of benefits of vaccine and cues to action were both strongly associated with vaccination. Physician or healthcare worker recommendation has been shown to be highly influential in making healthcare-related decisions, especially in Thailand [27–29]. Additionally, in the Thai influenza vaccination program context, where vaccine is available for a short period of time and distributed on a first-come, first-serve basis, being informed that the vaccine is available, through healthcare workers or media, may be enough to prompt vaccination. Among those not vaccinated, 28% reported not knowing the vaccine was available. Expanding publicity around the annual vaccination campaigns may help to increase coverage rates, in addition to improving vaccine supply, ensuring equitable distribution and increasing awareness about benefits of influenza vaccine.

Our evaluation has some limitations. Attitudes towards vaccination were assessed retrospectively, and there may have been differential recall between vaccinated and unvaccinated participants. VES has not been validated as a predictor of mortality (i.e. vulnerability) in Thailand, and it is possible that VES may not adequately reflect functional status of older Thai adults. However, we examined several outcomes related to functional status and none was a significant predictor of vaccination in multivariable models. Our sample was taken from just one out of 76 provinces of Thailand and may not be generalizable to all of Thailand. In addition, we evaluated vaccination after an enhanced campaign that purposefully targeted an increase in vaccination among persons ≥65 years old. Although vaccination rate after the enhanced campaign was no different than the self-reported vaccination rate for the previous year, the enhanced campaign may have reached individuals who otherwise would not have been vaccinated.

## Conclusion

In conclusion, distance to vaccination center, perceived benefits and cues to action, but not functional status, were associated with influenza vaccination of Thai adults ≥65 years. These data suggest that reducing the distance to vaccination clinics, perhaps through the use of mobile clinics and health centers, improving communication around benefits of vaccination to public and providers, and publicizing the availability of influenza vaccine could increase vaccination coverage among older adults in Thailand.

## Supporting information

S1 Table Questions used to develop Health Belief Model (HBM) constructs and scoring. (DOCX)

S2 Table Numbers and percentages to responses and scored components of the modified Vulnerable Elders Survey [15]. (DOCX)

S3 Table P-values calculated from Rao-Scott Chi square tests for association between demographic and functional status categorical variables. *Chi square test could not be calculated due to cells with 0 observations. Shaded observations are those considered statistically significant.
(DOCX)

S4 Table Reasons for not getting influenza vaccine in 2014 (n = 365). (DOCX)

S1 Dataset (XLSX)

S1 Codebook (DOCX)

## Abbreviations

VE: Vaccine Effectiveness
VES: Vulnerable Elders Survey
MOPH: Ministry of Public Health
NHSO: National Health Security Organization
HBM: Health Belief Model
