# Supplementary material for: Predictors of seasonal influenza vaccination among older adults in Thailand
Source: PLoS One. 2017 Nov 29;12(11):e0188422. doi: 10.1371/journal.pone.0188422 (PMC5706686; doi:10.1371/journal.pone.0188422)
Supplement: S1 Table — (DOCX) [file pone.0188422.s001.docx]

**S1 Table. Questions used to develop Health Belief Model (HBM) constructs and scoring**

| **HBM construct** | **Wording of questions** | **Scoring** | **Range of possible values for construct** |
| --- | --- | --- | --- |

| Susceptibility | How likely are you to get sick with influenza? | 1=Unlikely  2=Neither likely nor unlikely  3= Likely | 2 – 6 |
| --- | --- | --- | --- |
|  | How likely is someone in your family or group of friends to get sick with influenza? | 1=Unlikely  2=Neither likely nor unlikely  3= Likely |  |
| Severity | How likely are you to get severely sick (require hospitalization) with influenza? | 1=Unlikely  2=Neither likely nor unlikely  3= Likely | 3 – 9 |
|  | How likely is someone in your family or group of friends to get severely sick (require hospitalization) with influenza? | 1=Unlikely  2=Neither likely nor unlikely 3= Likely |  |
|  | How likely it is that influenza will disrupt the hospitals and clinics in your area (for example, overcrowded hospitals?) | 1=Unlikely  2=Neither likely nor unlikely 3= Likely |  |
| Benefits | How good or bad do you think the influenza vaccine shot is in preventing someone from becoming ill with influenza? | 1= Bad  2=Neither bad nor good 3=Good | 1 – 3 |
| Barriers | Ever experienced side effects from vaccination | 0=No 1=Yes | 1 – 4 |
|  | How safe do you think the influenza vaccine is? | 1=Safe  2=Neither safe nor unsafe 3=Unsafe |  |
| Cues to action | In the last three months, did a doctor, nurse or healthcare worker recommend that you get an influenza vaccination? | 0=No 1=Yes | 0 – 4 |
|  | In the last three months, did a friend or relative recommend that you get an influenza vaccination? | 0=No 1=Yes |  |
|  | In the last three months, have you heard anyone give a health message about influenza or the influenza vaccine, on the radio, television or in person? | 0=No 1=Yes |  |
|  | In the last three months, have you seen any posters or flyers on influenza or the influenza vaccine? | 0=No 1=Yes |  |
