# Supplementary material for: Predictors of seasonal influenza vaccination among older adults in Thailand
Source: PLoS One. 2017 Nov 29;12(11):e0188422. doi: 10.1371/journal.pone.0188422 (PMC5706686; doi:10.1371/journal.pone.0188422)
Supplement: S2 Table — (DOCX) [file pone.0188422.s002.docx]

**S2 Table. Numbers and percentages to responses and scored components of the modified Vulnerable Elders Survey [15]**

| **Question** | **Response** | **N (%)** | **Score component** | **Scoring algorithm** | **Scores** | **N (%)** |
| --- | --- | --- | --- | --- | --- | --- |
| Age | 65-74  75-84  >85 | 383 (67)  164 (28)  33 (5) | Age | 1 point for age 75-84  3 points for age >85 | 0  1  3 | 384 (67)  164 (28)  33 (5) |
| Health self-assessment | Poor  Fair  Good  Very good or Excellent  Don’t know | 44 (7)  183 (32)  213 (38)  92 (15)  49 (7) | Health  (Missing=49) | 1 point for ‘fair’ or ‘poor’ | 0  1 | 305 (57)  227 (43) |
| What is your average difficulty with the following activities: | | | Physical activity (Missing=3) | 1 point each for ‘a lot’ or ‘unable to do’; maximum of 2 points | 0  1  2 | 418 (73)  68 (12)  92 (15) |
| Stooping, crouching or kneeling | None  A little  Some  A lot  Unable to do  No answer | 255 (44)  156 (27)  100 (18)  52 (8)  18 (3)  0 (0) |  |  |  |  |
| Lifting or carrying objects as heavy as 5 kilos? | None  A little  Some  A lot  Unable to do  No answer | 329 (57)  79 (13)  60 (11)  49 (8)  60 (10)  4 (1) |  |  |  |  |
| Writing or handling and grasping small objects? | None  A little  Some  A lot  Unable to do  No answer | 463 (81)  76 (12)  21 (4)  16 (3)  5 (1)  0 (0) |  |  |  |  |
| Walking a half of a kilometer | None  A little  Some  A lot  Unable to do  No answer | 368 (62)  75 (14)  46 (9)  60 (11)  31 (4)  1 (0) |  |  |  |  |
| Heavy housework such as scrubbing floors or washing windows | None  A little  Some  A lot  Unable to do  No answer | 351 (59)  101 (18)  39 (7)  35 (7)  34 (5)  21 (4) |  |  |  |  |
| Because of your health or a physical condition, do you have any difficulty: | | | Needing help | 4 points for one or more responses indicating either ‘help’ with the activity or the activity was not done because of health reasons. | 0  4 | 478 (84)  103 (16) |
| Shopping for personal items (like medications or toilet items)? | Yes  No  Don’t do  Declined to answer | 61 (10)  390 (67)  130 (23)  0 (0) |  |  |  |  |
| If yes, do you get help with shopping? | Yes  No  Declined to answer | 59 (94)  2 (6)  0 (0) |  |  |  |  |
| If you don’t shop, is that because of your health? | Yes  No  Declined to answer | 37 (26)  93 (74)  0 (0) |  |  |  |  |
| Managing money (like keeping track of expenses or paying bills)? | Yes  No  Don’t do  Declined to answer | 33 (4)  475 (83)  73 (13)  0 (0) |  |  |  |  |
| If yes, do you get help with managing money? | Yes  No  Declined to answer | 30 (87)  3 (13)  0 (0) |  |  |  |  |
| If you don’t manage your money, is that because of your health? | Yes  No  Declined to answer | 18 (19)  55 (81)  0 (0) |  |  |  |  |
| Walking across the room? *Use of cane or walker is okay.* | Yes  No  Don’t do  Declined to answer | 19 (2)  562 (98)  0 (0)  0 (0) |  |  |  |  |
| If yes, do you get help with walking? | Yes  No  Declined to answer | 13 (60)  6 (40)  0 (0) |  |  |  |  |
| If you can’t walk across the room, is that because of your health? | Yes  No  Declined to answer | N/A |  |  |  |  |
| Bathing or showering? | Yes  No  Don’t do  Declined to answer | 15 (2)  565 (98)  1 (0)  0 (0) |  |  |  |  |
| If yes, do you get help with bathing or showering? | Yes  No  Declined to answer | 15 (100)  0 (0)  0 (0) |  |  |  |  |
| If you can’t bathe or shower, is that because of your health? | Yes  No  Declined to answer | 1 (100)  0 (0)  0 (0) |  |  |  |  |
