# Supplementary material for: Predictors of seasonal influenza vaccination among older adults in Thailand
Source: PLoS One. 2017 Nov 29;12(11):e0188422. doi: 10.1371/journal.pone.0188422 (PMC5706686; doi:10.1371/journal.pone.0188422)
Supplement: S3 Table — *Chi square test could not be calculated due to cells with 0 observations. Shaded observations are those considered statistically significant. (DOCX) [file pone.0188422.s003.docx]

**S3 Table. *P*-values calculated from Rao-Scott Chi square tests for association between demographic and functional status categorical variables.**

| Variable | Sex | Marital status | Education | Income | Wealth | District | Distance | Physician visit | Hospital admission | Chronic disease | Not go out daily | Fall down <6 m | Memory loss | Vulnerable |
| --- | --- | --- | --- | --- | --- | --- | --- | --- | --- | --- | --- | --- | --- | --- |
| Age | 0.24 | <0.0001 | 0.0008 | 0.20 | 0.43 | 0.11 | 0.0027 | 0.03 | 0.46 | 0.50 | 0.0042 | 0.74 | 0.32 | * |
| Sex |  | <0.0001 | <0.0001 | 0.83 | 0.26 | 0.57 | 0.27 | 0.0001 | 0.77 | 0.29 | 0.01 | 0.10 | 0.71 | 0.0002 |
| Marital status |  |  | 0.02 | 0.89 | 0.63 | 0.53 | 0.34 | 0.14 | 0.04 | 0.23 | <0.0001 | 0.30 | 0.04 | <0.0001 |
| Education |  |  |  | <0.0001 | * | 0.02 | 0.33 | 0.90 | 0.52 | 0.0003 | <0.0001 | 0.20 | 0.0014 | 0.0098 |
| Income |  |  |  |  | <0.0001 | 0.23 | 0.0091 | 0.99 | 0.80 | 0.22 | 0.11 | 0.12 | 0.18 | 0.56 |
| Wealth |  |  |  |  |  | <0.0001 | 0.0007 | 0.07 | 0.93 | 0.08 | 0.0047 | 0.16 | 0.0048 | 0.47 |
| District |  |  |  |  |  |  | * | 0.73 | 0.81 | 0.93 | 0.25 | 0.63 | 0.01 | 0.12 |
| Distance |  |  |  |  |  |  |  | 0.82 | 0.38 | 0.26 | 0.10 | 0.63 | 0.0056 | <0.0001 |
| Physician visit |  |  |  |  |  |  |  |  | 0.0007 | <0.0001 | 0.42 | 0.0085 | 0.33 | 0.89 |
| Hospital admission |  |  |  |  |  |  |  |  |  | 0.0003 | 0.24 | 0.61 | 0.39 | 0.05 |
| Chronic disease |  |  |  |  |  |  |  |  |  |  | 0.0066 | 0.32 | 0.50 | 0.04 |
| Not go out daily |  |  |  |  |  |  |  |  |  |  |  | 0.08 | 0.04 | <0.0001 |
| Fall down <6 m |  |  |  |  |  |  |  |  |  |  |  |  | 0.02 | 0.24 |
| Memory loss |  |  |  |  |  |  |  |  |  |  |  |  |  | <0.0001 |

*Chi square test could not be calculated due to cells with 0 observations. Shaded observations are those considered statistically significant.
