# Supplementary material for: Predictors of seasonal influenza vaccination among older adults in Thailand
Source: PLoS One. 2017 Nov 29;12(11):e0188422. doi: 10.1371/journal.pone.0188422 (PMC5706686; doi:10.1371/journal.pone.0188422)
Supplement: S4 Table — (DOCX) [file pone.0188422.s004.docx]

**S4 Table. Reasons for not getting influenza vaccine in 2014 (n=365)**

| Reasons | n (%) |
| --- | --- |
| Vaccine is not available/Not present/Unable to purchase | 101 (28) |
| I’m not susceptible to flu | 54 (15) |
| Did not consider it | 24 (7) |
| Flu vaccine is not safe | 17 (5) |
| Don’t know | 16 (4) |
| No means to get to vaccination center | 7 (2) |
| Flu is not severe | 7 (2) |
| Vaccine is not effective | 0 (0) |
