# Supplementary material for: Predictors of seasonal influenza vaccination among older adults in Thailand
Source: PLoS One. 2017 Nov 29;12(11):e0188422. doi: 10.1371/journal.pone.0188422 (PMC5706686; doi:10.1371/journal.pone.0188422)
Supplement: S1 Codebook — (DOCX) [file pone.0188422.s006.docx]

**Code Book**

**Flu 7**

| **Variable name** | **Type** | **Question** | **Codes** | **Comments** |
| --- | --- | --- | --- | --- |
| qid | Text | Study identification number |  |  |
| village | Text | Village | 48010401 Nongbua  48010104 Chumchon Nong Beuk Thung  48011009 Thako Tai  48011411 Nongsang  48011212 Comchon Wat Inpeng  48010621 Wung Krasae  48011603 Chumchon Nong Beuk Tha  48010131 Chumchon Wat Phosri  48010808 Chanod  48010510 Phrayod Mueang Kwang  48011118 Phonsawan  48020202 Kungkone  48020106 Bankok  48020807 Nachueknoi  48020603 Nongbua  48020310 Nadokmai  48080101 Nongbuatao  48080206 Na In  48080501 Thabosongkram  48080610 Bankha  48080810 Phonsawang  48080314 Kor  48050405 Hluksilatai  48050207 Bantong Nue  48050602 Sanphun tung  48050705 Nonsa-Ard  48051107 Kudchimnoi  48050108 Photong  48051010 Banmaiphosri  48050811 Bannakumtai |  |
| invresult_01 | N | Outcome of first attempted visit | 1 Interview completed  2 Interview partially completed – will return to finish  3 Interview partially completed – unable to return to finish  4 Refused  5 Ineligible  6 Absent and unlikely to return in next 7 days  7 Absent temporarily – will try again  8 Moved within study area – will try to contact  9 Moved outside of study area – cannot contact  10 Died. Provide date of death  11 No one knows the participant/Cannot be located  12 Participant cannot communicate  77 Return to collect data again/continue |  |
| invresult_02 | N | Outcome of second attempted visit | 1 Interview completed  2 Interview partially completed – will return to finish  3 Interview partially completed – unable to return to finish  4 Refused  5 Ineligible  6 Absent and unlikely to return in next 7 days  7 Absent temporarily – will try again  8 Moved within study area – will try to contact  9 Moved outside of study area – cannot contact  10 Died. Provide date of death  11 No one knows the participant/Cannot be located  12 Participant cannot communicate  77 Return to collect data again/continue |  |
| invresult_03 | N | Outcome of third attempted visit | 1 Interview completed  2 Interview partially completed – will return to finish  3 Interview partially completed – unable to return to finish  4 Refused  5 Ineligible  6 Absent and unlikely to return in next 7 days  7 Absent temporarily – will try again  8 Moved within study area – will try to contact  9 Moved outside of study area – cannot contact  10 Died. Provide date of death  11 No one knows the participant/Cannot be located  12 Participant cannot communicate  77 Return to collect data again/continue |  |
| iscomplete | Text | Is the data complete? | Yes  No |  |
| dod | Text | Date of death |  |  |
| selfanswer | N | Can participant respond on his or her own or will questions be asked solely of a caregiver? | 1 Respond on his or her own  2 Mixed  3 Responses only from caregiver |  |
| dob | Text | What is your date of birth? | Yyyymmdd  4 Do not know  99 Unknown | If month and day are unknown, 1 May was used. If year was unknown, calculated from age. |
| dob_aprx | Text | Calculated date of birth |  |  |
| elderly_inc_where | N | Where do you receive your monthly government stipend? | 1 Within this district  2 Outside of this district but within Nakhon Phanom Province  3 Outside of Nakhon Phanom Province  4 Does not receive a stipend  77 Does not receive a stipend |  |
| mostlive_where | N | If no stipend is received, where did you spend most of your time since Songkran? | 1 Within this district  2 Outside of this district but within Nakhon Phanom Province  3 Outside of Nakhon Phanom Province  4 Do not know  4 Do not know |  |
| consent | N | Participant or participant’s caregiver have read and signed the informed consent form? | 1 Yes  2 No |  |
| notjoin_reason | Text | please specify the reason for refusal |  |  |
| flu_known | N | Have you ever heard of ‘influenza’ before? | 1 Yes  2 No  3 Do not know  4 Declined to answer |  |
| What is ‘influenza’? *Listen to the answers and check all that are mentioned. When the participant has finished responding, prompt once for any additional answers.* | | | | |
| flu_is_a | N | Disease | 1 Yes  2 No |  |
| flu_is_b | N | Respiratory disease | 1 Yes  2 No |  |
| flu_is_c | N | Stomach disease | 1 Yes  2 No |  |
| flu_is_d | N | Virus | 1 Yes  2 No |  |
| flu_is_e | N | Bacteria | 1 Yes  2 No |  |
| flu_is_f | N | Other | 1 Yes  2 No |  |
| flu_is_g | N | Do not know | 1 Yes  2 No |  |
| flu_is_h | N | Declined to answer | 1 Yes  2 No |  |
| flu_risk_self | N | Are you likely or unlikely to get sick with influenza? | 1 Likely  2 Neither likely nor unlikely  3 Unlikely  4 Do not know  5 Declined to answer |  |
| flu_risk_surround | N | How likely is someone in your family or group of friends to get sick with influenza? | 1 Likely  2 Neither likely nor unlikely  3 Unlikely  4 Do not know  5 Declined to answer |  |
| flu_risk_ipd_self | N | How likely are you to get severely sick (require hospitalization) with influenza? | 1 Likely  2 Neither likely nor unlikely  3 Unlikely  4 Do not know  5 Declined to answer |  |
| flu_risk_ipd_surround | N | How likely is someone in your family or group of friends to get severely sick (require hospitalization) with influenza? | 1 Likely  2 Neither likely nor unlikely  3 Unlikely  4 Do not know  5 Declined to answer |  |
| flu_effect_hospital | N | How likely is it that influenza will disrupt the hospitals and clinics in your area (for example, overcrowded hospitals?) | 1 Likely  2 Neither likely nor unlikely  3 Unlikely  4 Do not know  5 Declined to answer |  |
| personalhealth | N | Since this past June, have you seen a doctor or other health care professional about your own health at a doctor's office, a clinic or some other place? | 1 Yes  2 No  3 Do not know  4 Declined to answer |  |
| ever_fshot | N | An influenza vaccine shot is usually given between May and September and protects against influenza for the influenza season. Have you ever had an influenza vaccine shot? | 1 Yes  2 No  3 Do not know  4 Decline |  |
| fhot_songk | N | Since Songkran this year, have you had an influenza vaccine shot? | 1 Yes  2 No  4 Do not know  99 Decline | Missing for those who said no to ever_fshot |
| fshot_songk_w | N | If yes, where did you get the influenza vaccine shot? | 1 Provincial hospital  2 District hospital  3 Private doctor’s office  4 Mobile clinic  5 Elsewhere  5 Do not know  6 Decline | Missing if said no to fhot_songk |
| fshot_songk_w_hosp | Text | Name of hospital | 10000 Nakhonphanom hospital  20000 Sakhonnakhon hospital  30000 Srinagarind hospital  40000 Udornthani hospital  10001 Plapak hospital  10002 Renunakorn hospital  10003 Nakae hospital  10004 Thatphanom hospital  10005 Tha U-tane hospital  10006 Phonesawan hospital  10007 Srisongkram hospital  10008 Nawa hospital  10009 Banphaeng hospital  10010 Nathom hospital  10011 Military hospital  48101 Huaphon  48102 Na Rat Kwai  48103 Kuruku  48104 Ban Phung  48105 Na Mon  48106 Nongpladuk  48107 Ban Hom  48108 A-Samard  48109 kham Thao  48110 Cha ngom  48111 Chanod  48112 Banklang  48113 Dongtew  48114 Nongchan  48115 Tha Kho  48116 Na Luang  48117 Kham Toei  48118 Dondang  48119 Thung Mon  48120 Nong Yat  48121 Ban Kum Pok  48122 Ban Bua  48123 Dong Khwang  48124 Chok Am Nuai  48125 Sukasem  48201 Nong Hi  48202 Kuta Kai  48203 Nadokmai  48204 Koksawang  48205 Koksung  48206 Mahar Chai  48207 Na Makua  48208 Phon SW  48209 Nong Thao Yai  48501 Fang Daeng  48502 Phone Phaeng  48503 Tan Kud  48504 Pha Klang Tung  48505 Na Thon  48506 Dong Yo  48507 San Phan  48508 Don Nahong  48509 Nam Kham  48510 Ban Tu  48511 Sai Moon  48512 Um Mao  48513 Na Nhad  48514 Khud Chim  48515 Koksawang P  48801 Na Dua  48802 E-ud  48803 Nong Puae  48804 Seangsao  48805 Bankae  48806 Pakyam  48807 Sam Phong  48808 Tha Bo  48809 Don Samo  48810 Ban Kha  48811 Kham Bea Yai  48812 Ban Lao  48813 Na kum  48814 Phu Kratae  48815 Phone Sawang  48816 Na Pho  48817 Siew -SK  48818 Hat Phaeng | Provincial hospital  Provincial hospital  Provincial hospital  Provincial hospital  District hospital  District hospital  District hospital  District hospital  District hospital  District hospital  District hospital  District hospital  District hospital  District hospital  District hospital  Sub-district hospital  Sub-district hospital  Sub-district hospital  Sub-district hospital  Sub-district hospital  Sub-district hospital  Sub-district hospital  Sub-district hospital  Sub-district hospital  Sub-district hospital  Sub-district hospital  Sub-district hospital  Sub-district hospital  Sub-district hospital  Sub-district hospital  Sub-district hospital  Sub-district hospital  Sub-district hospital  Sub-district hospital  Sub-district hospital  Sub-district hospital  Sub-district hospital  Sub-district hospital  Sub-district hospital  Sub-district hospital  Sub-district hospital  Sub-district hospital  Sub-district hospital  Sub-district hospital  Sub-district hospital  Sub-district hospital  Sub-district hospital  Sub-district hospital  Sub-district hospital  Sub-district hospital  Sub-district hospital  Sub-district hospital  Sub-district hospital  Sub-district hospital  Sub-district hospital  Sub-district hospital  Sub-district hospital  Sub-district hospital  Sub-district hospital  Sub-district hospital  Sub-district hospital  Sub-district hospital  Sub-district hospital  Sub-district hospital  Sub-district hospital  Sub-district hospital  Sub-district hospital  Sub-district hospital  Sub-district hospital  Sub-district hospital  Sub-district hospital  Sub-district hospital  Sub-district hospital  Sub-district hospital  Sub-district hospital  Sub-district hospital  Sub-district hospital  Sub-district hospital  Sub-district hospital  Sub-district hospital  Sub-district hospital  Sub-district hospital |
| fshot_lyear | N | Did you get an influenza vaccine shot last year (2013)? | 1 Yes  2 No  3 Do not know  4 Decline |  |
| fshot_songk_med_persd | N | Since this past Songkran, did a doctor, nurse or healthcare worker recommend that you get an influenza vaccination? | 1 Yes  2 No  3 Do not know  4 Decline |  |
| fshot_songk_persd_a | N | Physician | 1 Yes  2 No |  |
| fshot_songk_persd_b | N | Nurse | 1 Yes  2 No |  |
| fshot_songk_persd_c | N | Health volunteer | 1 Yes  2 No |  |
| fshot_songk_persd_d | N | Other healthcare worker | 1 Yes  2 No |  |
| fshot_june_nonmed_persd | N | Since this past June, did a friend or relative recommend that you get an influenza vaccination? | 1 Yes  2 No  3 Do not know  4 Decline |  |
| flu_songk_info | N | Since this past Songkran, have you heard anyone give a health message about influenza or the influenza vaccine, on the radio, television or in person? | 1 Yes  2 No  3 Do not know  4 Decline |  |
| flu_songk_info_by_a | N | Message on radio | 1 Yes  2 No |  |
| flu_songk_info_by_b | N | Message on loudspeaker in village | 1 Yes  2 No |  |
| flu_songk_info_by_c | N | Message on loudspeaker at hospital | 1 Yes  2 No |  |
| flu_songk_info_by_d | N | Message on television or cable | 1 Yes  2 No |  |
| flu_songk_info_by_e | N | In person health talk | 1 Yes  2 No |  |
| flu_songk_media | N | Since this past Songkran, have you seen any posters or flyers on influenza or the influenza vaccine? | 1 Yes  2 No  3 Do not know  4 Decline |  |
| flu_songk_media_by_a | N | Poster | 1 Yes  2 No |  |
| flu_songk_media_by_b | N | Billboard | 1 Yes  2 No |  |
| flu_songk_media_by_c | N | Flyers or small signs | 1 Yes  2 No |  |
| flu_songk_media_by_d | N | Computer/internet | 1 Yes  2 No |  |
| flu_vac_effective | N | How good or bad do you think the influenza vaccine shot is in preventing someone from getting sick with influenza? | 1 Good  2 Neither good nor bad  3 Bad  4 Do not know  5 Declined to answer |  |
| flu_vac_safety | N | How safe do you think the influenza vaccine is? Would you say very safe, somewhat safe, somewhat unsafe or very unsafe? | 1 Safe  2 Neither safe nor unsafe  3 Unsafe  4 Do not know  5 Declined to answer |  |
| What age groups do you think are at the greatest risk of severe disease from influenza infection? | | | | |
| flu_age_risk_a | N | Infants | 1 Yes  2 No |  |
| flu_age_risk_b | N | Toddlers | 1 Yes  2 No |  |
| flu_age_risk_c | N | School-age children | 1 Yes  2 No |  |
| flu_age_risk_d | N | Mature adults | 1 Yes  2 No |  |
| flu_age_risk_e | N | Persons over age 65 | 1 Yes  2 No |  |
| flu_age_risk_f | N | No one more at risk than others | 1 Yes  2 No |  |
| flu_age_risk_g | N | Other | 1 Yes  2 No |  |
| flu_age_risk_h | N | Do not know | 1 Yes  2 No |  |
| flu_age_risk_i | N | Declined to answer | 1 Yes  2 No |  |
| flu_age_risk_oth | Text | Other, please specify…… |  |  |
| What was or were the reasons you did not get an influenza vaccine shot this year? | | | | |
| nonvac_why_lyear_a | N | I have never considered it before | 1 Yes  2 No | Missing for those who got a shot |
| nonvac_why_lyear_b | N | I didn’t know it was available | 1 Yes  2 No |  |
| nonvac_why_lyear_c | N | I don’t think the vaccine is effective enough | 1 Yes  2 No |  |
| nonvac_why_lyear_d | N | I don’t think I am very likely to catch the influenza | 1 Yes  2 No |  |
| nonvac_why_lyear_e | N | I don’t think influenza is a serious illness | 1 Yes  2 No |  |
| nonvac_why_lyear_f | N | I am afraid of the side effects | 1 Yes  2 No |  |
| nonvac_why_lyear_g | N | I have specific contraindications/allergies | 1 Yes  2 No |  |
| nonvac_why_lyear_h | N | I wanted to get vaccinated but I heard there was no vaccine available | 1 Yes  2 No |  |
| nonvac_why_lyear_i | N | I wanted to get vaccinated but the health center told me they had no more vaccine | 1 Yes  2 No |  |
| nonvac_why_lyear_j | N | I wanted to get vaccinated but had no way to the vaccination site | 1 Yes  2 No |  |
| nonvac_why_lyear_k | N | I was not in the area at the time (June) | 1 Yes  2 No |  |
| nonvac_why_lyear_l | N | I did not have the money for the vaccine | 1 Yes  2 No |  |
| nonvac_why_lyear_m | N | Other reason | 1 Yes  2 No | Very critical question to code answers to. |
| nonvac_why_lyear_n | N | Do not know | 1 Yes  2 No |  |
| nonvac_why_lyear_o | N | Declined to answer | 1 Yes  2 No |  |
| fshot_effect_ever | N | When you were vaccinated against influenza, did you ever have any bad effects from the vaccine? | 1 Yes  2 No  3 Do not know  4 Decline |  |
| fshot_effect_how | N | How serious were the bad effects? | 1 Mild and self-limiting  2 Moderate  3 Severe  4 Do not know  5 Declined to answer |  |
| Please describe the bad effects you had from the influenza vaccination. | | | | |
| fshot_effect_a | N | Headache | 1 Yes  2 No |  |
| fshot_effect_b | N | Muscle ache where the needle entered | 1 Yes  2 No |  |
| fshot_effect_c | N | Redness or irritation where the needle entered | 1 Yes  2 No |  |
| fshot_effect_d | N | Runny nose | 1 Yes  2 No |  |
| fshot_effect_e | N | Body aches | 1 Yes  2 No |  |
| fshot_effect_f | N | Dizziness | 1 Yes  2 No |  |
| fshot_effect_g | N | Fever | 1 Yes  2 No |  |
| fshot_effect_h | N | Tiredness | 1 Yes  2 No |  |
| fshot_effect_i | N | Other | 1 Yes  2 No |  |
| fshot_effect_j | N | Do not know | 1 Yes  2 No |  |
| fshot_effect_k | N | Declined to answer | 1 Yes  2 No |  |
| fshot_effect_oth | Text | Other, specify |  |  |
| your_health | N | In general, compared to other people your age, would you say that your health is poor, fair, good, very good or excellent? | 1 Very poor  2 Bad  3 Fair  4 Good  5 Very good  6 Do not know  7 Declined to answer |  |
| severe_sick_lyear | N | Have you experienced any severe illness in the last year? | 1 Yes  2 No  4 Do not know  5 Declined to answer |  |
| admit_lyear | N | Did you have to be hospitalized for any illness in the last year? | 1 Yes  2 No  3 Do not know  4 Declined to answer |  |
| falldown_6m | N | Did you fall during the last 6 months? | 1 Yes  2 No  3 Do not know  4 Declined to answer |  |
| falldown_6m_n | N | If so, how many times did you fall in the last 6 months? | Xx  1 Do not know  2 Declined to answer |  |
| Please tell us what types of health insurance you have. | | | | |
| health_insur_a | N | Universal coverage | 1 Yes  2 No |  |
| health_insur_b | N | Government health insurance | 1 Yes  2 No |  |
| health_insur_c | N | Private health insurance | 1 Yes  2 No |  |
| health_insur_d | N | Not eligible | 1 Yes  2 No |  |
| health_insur_e | N | Do not know | 1 Yes  2 No |  |
| health_insur_f | N | Declined to answer | 1 Yes  2 No |  |
| How much difficulty, on average, do you have with the following physical activities: | | | | |
| bend_down | N | Stooping, crouching or kneeling | 1 No difficulty  2 Little difficulty  3 Some difficulty  4 A lot of difficulty  5 Unable to do  6 Unable to answer |  |
| carry5kg | N | Lifting or carrying objects as heavy as 5 kilos? | 1 No difficulty  2 Little difficulty  3 Some difficulty  4 A lot of difficulty  5 Unable to do  6 Unable to answer |  |
| writing | N | Writing or handling and grasping small objects? | 1 No difficulty  2 Little difficulty  3 Some difficulty  4 A lot of difficulty  5 Unable to do  6 Unable to answer |  |
| walk500m | N | Walking a half of a kilometer | 1 No difficulty  2 Little difficulty  3 Some difficulty  4 A lot of difficulty  5 Unable to do  6 Unable to answer |  |
| housework | N | Heavy housework such as scrubbing floors or washing windows | 1 No difficulty  2 Little difficulty  3 Some difficulty  4 A lot of difficulty  5 Unable to do  6 Unable to answer |  |
| shopping_self | N | Because of your health or a physical condition, do you have any difficulty shopping for personal items (like medications or toilet items)? | 1 Yes  2 No  3 Don’t do  4 Do not know  5 Decline |  |
| shopping_asst | N | If yes, do you get help? | 1 Yes  2 No  3 Decline |  |
| shopping_health | N | If no, is that because of your health? | 1 Yes  2 No  3 Decline |  |
| finance_self | N | Because of your health or a physical condition, do you have any difficulty managing money (like keeping track of expenses or paying bills)? | 1 Yes  2 No  3 Don’t do  4 Do not know  5 Decline |  |
| finance_asst | N | If yes, do you get help? | 1 Yes  2 No  3 Decline |  |
| finance_health | N | If no, is that because of your health? | 1 Yes  2 No  3 Decline |  |
| move_self | N | Because of your health or a physical condition, do you have any difficulty walking across the room? | 1 Yes  2 No  3 Don’t do  4 Do not know  5 Decline |  |
| move_asst | N | If yes, do you get help? | 1 Yes  2 No  3 Decline |  |
| move_health | N | If no, is that because of your health? | 1 Yes  2 No  3 Decline |  |
| bath_self | N | Because of your health or a physical condition, do you have any difficulty bathing or showering? | 1 Yes  2 No  3 Don’t do  4 Do not know  5 Decline |  |
| bath_asst | N | If yes, do you get help? | 1 Yes  2 No  3 Decline |  |
| bath_health | N | If no, is that because of your health? | 1 Yes  2 No  3 Decline |  |
| go_out_freq | N | How often do you leave your house for any reason (work, errands, exercise or to visit friends and family, etc.)? | 1 Rarely or never  2 About once a month  3 A few times a month  4 About once a week  5 A few times a week  6 Every day  7 Do not know  8 Declined to answer |  |
| memory | N | How would you rate your memory? | 1 Significant memory loss, confusion or dementia  2 Moderate memory loss, confusion or dementia  3 Minor memory loss  4 No memory problems  5 Do not know  6 Declined to answer |  |
| *Now I would like to ask you about your health history. Please tell me whether you (or your family member) have been diagnosed by a physician with any of the following conditions:* | | | | |
| lung_sick_ever | N | Chronic lung disease | 1 Yes  2 No  3 Do not know  4 Decline |  |
| lung_sick_a | N | COPD | 1 Yes  2 No |  |
| lung_sick_b | N | Emphysema | 1 Yes  2 No |  |
| lung_sick_c | N | Asthma | 1 Yes  2 No |  |
| lung_sick_d | N | Fibrosis | 1 Yes  2 No |  |
| lung_sick_e | N | Chronic bronchitis | 1 Yes  2 No |  |
| lung_sick_f | N | Tuberculosis | 1 Yes  2 No |  |
| lung_sick_g | N | Lung cancer | 1 Yes  2 No |  |
| lung_sick_h | N | Other | 1 Yes  2 No |  |
| lung_sick_i | N | Do not know | 1 Yes  2 No |  |
| lung_sick_j | N | Declined to answer | 1 Yes  2 No |  |
| lung_sick_oth | Text | Other, specify |  |  |
| heart_sick_ever | N | Chronic heart and circulatory disease | 1 Yes  2 No  3 Do not know  4 Decline |  |
| heart_sick_a | N | Hypertension | 1 Yes  2 No |  |
| heart_sick_b | N | Cardiomyopathy | 1 Yes  2 No |  |
| heart_sick_c | N | Coronary artery disease | 1 Yes  2 No |  |
| heart_sick_d | N | Heart valve disease | 1 Yes  2 No |  |
| heart_sick_e | N | Abnormal heart rhythms or arrhythmias | 1 Yes  2 No |  |
| heart_sick_f | N | Other | 1 Yes  2 No |  |
| heart_sick_g | N | Do not know | 1 Yes  2 No |  |
| heart_sick_h | N | Declined to answer | 1 Yes  2 No |  |
| stroke_sick | N | Cerebrovascular disease (stroke) | 1 Yes  2 No  3 Do not know  4 Decline |  |
| kidney_sick | N | Chronic kidney disease | 1 Yes  2 No  3 Do not know  4 Decline |  |
| liver_sick | N | Chronic liver disease | 1 Yes  2 No  3 Do not know  4 Decline |  |
| nerve_muscle_sick | N | Neurologic/neuromuscular disorder (including muscular dystrophy, cerebral palsy) | 1 Yes  2 No  3 Do not know  4 Decline |  |
| blood_sick_ever | N | Hemoglobinopathy, including thalassemia | 1 Yes  2 No  3 Do not know  4 Decline |  |
| blood_sick_a | N | Thalassemia | 1 Yes  2 No |  |
| blood_sick_b | N | Other | 1 Yes  2 No |  |
| blood_sick_c | N | Do not know | 1 Yes  2 No |  |
| blood_sick_d | N | Declined to answer | 1 Yes  2 No |  |
| metabolism_sick | N | Metabolic disease, including diabetes | 1 Yes  2 No  3 Do not know  4 Decline |  |
| metabolism_a | N | Diabetes | 1 Yes  2 No |  |
| metabolism_b | N | Other | 1 Yes  2 No |  |
| metabolism_c | N | Do not know | 1 Yes  2 No |  |
| metabolism_d | N | Declined to answer | 1 Yes  2 No |  |
| immune_sick | N | Do you have any immunosuppressive conditions (HIV, chemotherapy) | 1 Yes  2 No  3 Do not know  4 Decline |  |
| immune_a | N | Chemotherapy | 1 Yes  2 No |  |
| immune_b | N | Other | 1 Yes  2 No |  |
| immune_c | N | Do not know | 1 Yes  2 No |  |
| immune_d | N | Declined to answer | 1 Yes  2 No |  |
| immune_oth | Text | Other, specify |  |  |
| autoimmune_sick | N | Lupus | 1 Yes  2 No  3 Do not know  4 Decline |  |
| cancer_sick | N | Cancer, not reported above | 1 Yes  2 No  3 Do not know  4 Decline |  |
| sex | N | Sex | 1 Male  2 Female |  |
| marital | N | Are you currently married? | 1 Married  2 Widowed  3 Divorced/separated  4 Single (never married)  5 Do not know  6 Declined to answer  RECODED TO:  1 MARRIED  2 WIDOWED/DIVORCED/SEPARATED  4 SINGLE |  |
| live_in | N | Do you live in a group home or nursing facility for elderly? | 1 Yes  2 No |  |
| livewith_n | N | How many other people live with you in this house? |  |  |
| treat_where | N | If you become ill, where do you normally seek care? | 1 Provincial hospital  2 District hospital  3 Sub-district health center  4 Private physician  5 Don’t seek care  6 Other  7 Don’t know  8 Declined |  |
| edu | N | What is the highest level of education you completed? | 1 Never attended school  2 Some primary  3 Completed primary  4 Some secondary  5 Completed secondary  6 Some post-secondary education  7 Graduate degree  8 Do not know  9 Declined to answer |  |
| head_house | N | Are you the head of your household? | 1 Yes  2 No  3 Do not know  4 Declined |  |
| edu_head_house | N | If no, what is the highest level of education completed by the head of household? | 1 Never attended school  2 Some primary  3 Completed primary  4 Some secondary  5 Completed secondary  6 Some post-secondary education  7 Graduate degree  8 Do not know  9 Declined to answer |  |
| house_income | N | What is your average monthly household income in Thai baht? | 1 Less than 5,000  2 5,000-9,999  3 10,000-19,999  4 20,000-29,999  5 30,000-39,999  6 40,000 or more  7 Do not know  8 Declined to answer |  |
| Please tell us whether your (or your family member’s) household has any of the following items | | | | |
| homeappl_a | N | Motorcycle, moped, tuk tuk, etc | 1 Yes 2 No 77 DK |  |
| homeappl_b | N | Motor vehicle (car or pickup truck) | 1 Yes 2 No 77 DK |  |
| homeappl_c | N | Radio | 1 Yes 2 No 77 DK |  |
| homeappl_d | N | Television | 1 Yes 2 No 77 DK |  |
| homeappl_e | N | Refrigerator | 1 Yes 2 No 77 DK |  |
| homeappl_f | N | Computer | 1 Yes 2 No 77 DK |  |
| homeappl_g | N | Washing machine | 1 Yes 2 No 77 DK |  |
| homeappl_h | N | Jewelry | 1 Yes 2 No 77 DK |  |
| homeappl_i | N | Telephone (mobile or landline) | 1 Yes 2 No 77 DK |  |
| Useradio | N | Do you listen to the radio? | 1 Yes  2 No  3 Do not know  4 Declined |  |
| usetv | N | Do you watch television? | 1 Yes  2 No  3 Do not know  4 Declined |  |
| telephone_type_a | N | Mobile | 1 Yes  2 No |  |
| telephone_type_b | N | Landline | 1 Yes  2 No |  |
| telephone_type_c | N | Not sure | 1 Yes  2 No |  |
| telephone_type_d | N | Declined to answer | 1 Yes  2 No |  |
| telephone_use | N | Do you make and receive calls on the phone? | 1 Makes and receives calls  2 Makes calls only  3 Receives calls only  4 Neither makes nor receives calls  5 Do not know  6 Declined |  |
| house | N | *What kind of house does the participant live in?(Observe)* | 1 House (formal)  2 Informal setting |  |
| cooking_fuel | N | With what type of fuel do you normally use to cook, or someone else uses to cook for you? | 1 Electricity  2 Gas (propane, LPG)  3 Coal  4 Charcoal  5 Kerosene  6 Firewood or scrap wood  7 Other  8 Do not know  9 Declined to answer |  |
| drink_water | N | What is the main source of your drinking water? | 1 Piped water (government, private or communal)  2 Well/tube well  3 Bottled water  4 Pond, spring, river, lake  5 Other  6 Do not know  7 Declined to answer |  |
| lavatory | N | What type of toilet do you have? | 1 Flush toilet  2 Squat  3 Both flush and squat  4 Bucket toilet  5 None (outdoors)  6 Other  7 Do not know  8 Declined to answer |  |
| starving_lyear | N | Have you experienced any shortage of food in the last year? | 1 Yes  2 No |  |
| inv_by | N | Document who the main informant for this interview has been | 1 Participant  2 Main support person  3 Both |  |
| xcoor | N | Latitude |  |  |
| ycoor | N | Longitude |  |  |
| eligible | Text | Eligible | Yes  No |  |
| vaccinationstatus_2014 | N | Influenza vaccination status from NHSO database in year 2013 | 1 Received  2 Not received  3 No information |  |
| vaccination_place | Text | If vaccination status is received, where do you receive influenza vaccine? | Srisongkram hospital  Plapak hospital  Nakhonphanom hospital  Thatphanom hospital  Udornthani hospital |  |
|  |  |  |  |  |
